# YESdb: integrative analysis of environmental stress in yeast

**DOI:** 10.1093/database/baz023

**Published:** 2019-03-01

**Authors:** Evi Berchtold, Gergely Csaba, Ralf Zimmer

**Affiliations:** Institute of Bioinformatics, Department of Informatics, Ludwig-Maximilians-Universität München, Amalienstraße, München, Germany

## Abstract

The stress response in the model organisms *Saccharomyces cerevisiae* is a well-studied system for which many data sets are available. Already in 2000, it was discovered that yeast cells trigger a similar transcriptional response when different types of stress are applied. However, the exact regulatory mechanisms and differences between the different types of stress are still not understood.

Here, we present the Yeast Environmental Stress database (YESdb), a database containing all high-throughput experiments measuring various kinds of stress in yeast. The goal of the database is to allow the user to execute complex, integrative analyses of selected data sets, e.g. the comparison of measurements of the same stress using different platforms or differences between strains, stress strengths or types of stress. The analyses can be visualized in various ways and can be compiled into interactive reports to summarize and communicate the results.

The data sets are available as differential conditions (typically stressed vs control), which are grouped to time or concentration series when multiple measurements over time or concentrations are done in one experiment. An annotation ontology has been constructed to annotate the data sets with the type, duration and strength of the applied stress, the used strain and experimental platform as well as the publication date. These annotations can easily be combined to select all relevant data sets for an analysis.

YESdb allows to construct and execute Petri net-based workflows to perform predefined and custom analyses. E.g. to compare two types of stress (e.g. salt vs oxidative stress), the corresponding data sets are selected from the database, the consistently changed genes are defined and combined and the shared genes are characterized by enrichment analysis.

A broad collection of visualizations is available most of which are also interactive. The results of all analyses can be summarized in an interactive report. Visualizations of individual steps (transitions) of YESdb workflows can be automatically added to this report or customized visualizations as well as interpretive text can manually be added to the report.

Overall, YESdb aims at making all published data sets on yeast stress immediately available and comparable for integrated analysis of data sets and sets of genes in order to identify and assess hypotheses and mechanisms.

## Introduction

More and more high-throughput data are made publicly available in databases like GEO (2), ArrayExpress (15), SRA (14) or PRIDE (21). This published data can be used to complement newly measured data in various ways. Meta-analyses integrate diverse data sets from different studies, tissues or species to draw unbiased conclusions. While meta-analyses usually focus on data from the same or similar platforms, another way to benefit from published data is to integrate data sets from the same or a similar condition measured on different platforms (e.g. RNAseq and microarray data). Systematic biases of one platform can thus be identified and corrected for. Similarly, data sets that measure different levels (e.g. expression and protein levels) of the same condition can be combined to obtain a more complete picture of the changes in the cell.

Even though the integration of multiple data sets can improve the analysis, many studies ignore published data that could be integrated in their analysis. The first hurdle for integrative analyses is of course to find data that fit, which often involve reading detailed experimental descriptions to uncover how similar the conditions are. Furthermore, integrative analyses are often hindered by the need to preprocess the raw data that are stored in the public databases. Especially when the published data are measured on a different platform, a different preprocessing workflow has to be used.

To facilitate the use of published data, some databases offer analysis possibilities directly. GEO introduced the GEO2R tool, which allows one to use GEO data sets directly in R analyses. This is a very powerful tool but limited to users that are familiar with the R programming language. Other databases such as MEM (1) and SPELL (10) also allow the user to do some analyses directly on their website, but they focus mainly on co-expression studies.

Workflow managers (see (16) for a recent review) enable the user to conduct complicated pipelines to process the data. This allows the user to easily test the influence of parameter settings or the choice of specific methods. A major limitation for using a workflow manager for an integrative analysis is the search for and import of the already published data. Furthermore, pipelines are typically used for a standard analysis of the data (e.g. to derive the differentially expressed genes in an experiment), as the more specific downstream analyses cannot normally be re-used for another experiment, and the next step can often not be defined in advance, as it depends on the results of the previous step.

The stress response in *Saccharomyces cerevisiae* is an especially well-studied system for which many different data sets are available. However, there are still many unsolved questions of how the system is regulated for the different kinds of stress. To study the conserved and divergent parts of the system, an integrative analysis is needed.

yStreX (22) collected, classified and preprocessed several data sets measuring different stress conditions in yeast. It allows one to identify differentially expressed genes, to find conditions in which a gene is differentially expressed and enrichment analyses for single and multiple conditions. However, it has also several limitations: the collected data sets are required to have more than two replicates, so that many time series analyzing different kinds of stress with one replicate per time point are missing. Furthermore, it contains only gene expression data measured by microarrays, so that proteomics or sequencing data sets are not contained. This results in a total of 121 conditions, which is only a small subset of the available data.

We propose YESdb, a database that contains preprocessed differential expression data for various types of stress in the model organism *S. cerevisiae*. To make optimal use of the data, the database contains a Petri net-based workflow system, which allows the user to integrate multiple data sets. The results of the workflow are visualized in interactive reports, which contain a visual summary of each step in the workflow. Several runs of a workflow with different parameters can be directly compared in these reports. This way, the impacts of individual parameters in a complex analysis can easily be analyzed.

## Material and methods

### Data search strategy

To find the relevant data sets, the meta-data from GEO was filtered for data sets measuring RNA in *S. cerevisiae*, and the resulting data sets were searched for ‘treatment’/‘treated’, ‘adaptation’/‘adapted’, ‘exposure’/‘exposed’, ‘response’ and ‘stress’. This yielded 386 GEO Series of which most were microarray data sets contained in GEO and only 35 corresponded to high-throughput sequencing data sets contained in SRA. The same search terms were used to query ArrayExpress omitting data sets already contained in GEO. The resulting data sets were additionally manually filtered for relevance. For proteomics data, there are far fewer data sets available in PRIDE, which are unfortunately less standardized and less comprehensively annotated. Here we manually selected the relevant data sets for which MaxQuant (5) output was available and for which the individual conditions could be identified in the output.

**Figure 1:**
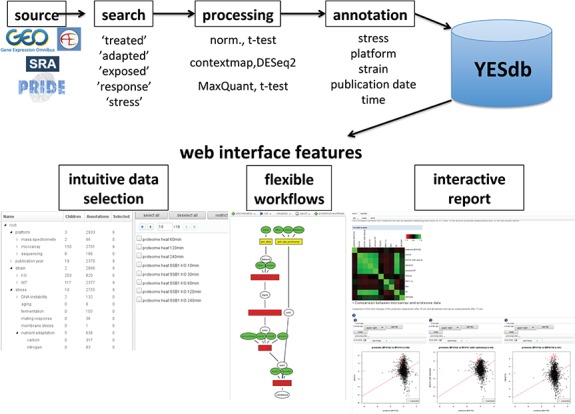
Overview of the data sources and web interface features of YESdb: All stress response data sets from GEO, ArrayExpress, SRA and PRIDE are selected, processed to differential conditions (‘DCs’) and annotated. The web interface features an intuitive data selection interface, workflows that allow to execute complex analyses and interactive reports to summarize and visualize the results.

### Data processing

YESdb contains already differential conditions (‘DCs’), so that the user does not have to identify replicates and the conditions that should be compared. To construct this configuration, we used a semi-automatic framework that first automatically identifies replicates and control/unstressed conditions, which are then manually corrected and completed. Additionally, time or concentration courses are saved as ‘series’, i.e. lists of ‘DCs’ together with the corresponding time or concentration.

For GEO, the data sets are already processed in most cases. A simple median normalization is used to make the individual samples comparable while not distorting the already processed (and normally normalized) values too much. The configurations are then used to calculate log$_2$ fold changes. When replicate raw intensities are available, t-tests between the case and control intensities are calculated and the resulting *p*-values are multiple testing corrected by the Benjamini–Hochberg correction. The SRA data sets are mapped by ContextMap (3), and differential expression was analyzed by DESeq (17). ArrayExpress does not contain processed data for all data sets. We thus used the R object stored in the database where it is available or used the ArrayExpress R-package (12) to load the data. The data sets that could be loaded like this were summarized by RMA (11) and fold changes and t-test *p*-values were calculated analogous to the GEO data sets. For PRIDE, SILAC fold changes of replicates are combined by taking the mean and log$_2$ fold changes and t-test *p*-values with Benjamini–Hochberg correction are calculated for LFQ data. In all cases, replicates and raw measurements are saved if available so that they can be used for visualization and filtering.

### Data annotation

We created an ontology of annotations to make it easy to find the relevant data sets for a specific analysis. This ontology contains the experimental platform, the publication date, the yeast strain that was used (including which genes were knocked out) and the kind of stress that was applied. Each GEO/ArrayExpress/SRA/PRIDE data set was manually mapped to all relevant terms in this ontology. To select the relevant data sets, we provide an easy-to-use interface where the ontology can be browsed and the ‘DCs’ or ‘series’ annotated to a selected term are shown. These entries can then be selected or excluded individually or all at once, and the entries selected so far can be restricted to those annotated in the current ontology term. This allows the user e.g. to select all ‘series’ annotated to heat shock at 37 and 39°C and restrict this selection to those ‘series’ that were measured by microarray and exclude all ‘series’ that used knockout strains.

Table 1 shows the first levels of this annotation hierarchy. The database contains 2933 ‘DCs’ and 392 ‘series’. Of these, 820 ‘DCs’ measure 203 different knockout strains and 2377 ‘DCs’ measure 117 different wild-type strains. Oxidative stress, osmotic stress, carbon source adaptation and temperature adaptation are the best-studied kinds of stress in our database, containing between 278 and 460 ‘DCs’.

**Table 1:** Overview of the annotations of the data sets contained in YESdb: Only the first level of annotation is shown, most annotations contain additional levels such as the specific platform or strain used or the strength of the applied stress; For the time annotation, only the most frequent entries are shown; The number of all (also indirect) child annotation terms are given in the last column; The data are processed to ‘DCs’ and (time or concentration) ‘series’

Annotation	DC	Series	Children
Platform	3612	512	162
Microarray	3380	480	152
Sequencing	186	29	8
Mass spectrometry	46	7	2
Publication year	3049	422	19
Strain	3545	504	351
Wild type	3012	428	123
Knock out	900	119	228
Stress	3426	495	76
Other	1428	205	16
Nutrient adaptation	730	104	5
Oxidative stress	466	57	20
Osmotic stress	361	57	19
Temperature	282	47	14
DNA instability	132	22	2
Fermentation	105	20	—
Mating response	40	7	—
Time	1644	—	124
30 min	293	—	—
60 min	149	—	—
2 h	190	—	—
20 min	97	—	—
15 min	76	—	—
10 min	68	—	—
5 min	59	—	—
...		

### Workflows

We implemented a Petri net-based workflow system to allow the user to easily perform integrative analyses of the data sets in the database. This system facilitates the identification of interesting genes from several data sets and to combine them in a flexible way to analyze different hypotheses. Table 2 shows an overview of the available transitions. There are transitions to define and combine sets of entities, for downstream analyses such as enrichment or simple network analysis and helper transitions to e.g. modify the ‘DCs’.

**Table 2:** Overview of workflow transitions: The first block of transitions defines ‘sets’ of interesting genes, the second block characterizes ‘sets’ of genes and the last block contains helper transitions

Name	Description
Set from DAG	Loads a list of ‘sets’ from a ‘DAG’, e.g. GO
Set from DiffCond	Defines a ‘set’ from a ‘DC’ by filtering the measurements (fold change, raw or *P*-value)
Binary set combination	Combines two ‘sets’ by set operations (intersect, difference and union)
Multi set combination	Combines multiple ‘sets’ by set operations
Count Filter	Defines a ‘set’ of the genes that are contained at least/most a given number of times in a list of ‘sets’
GetRegulators	Defines a ‘set’ of regulators from a ‘network’ that regulate at least one target from the given ‘set’
Enrichment	Calculates enrichment of a ‘set’ in a list of ‘sets’
Subnetwork	Extracts the subnetwork of a ‘set’ from a ‘network’
Reverse fold change	Swaps case and control conditions of a ‘DC’
Data set fold change	Generates a new ‘DC’ that is the fold change between two ‘DCs’ (e.g. DC1, stress1 vs control; DC2, stress2 vs control $\rightarrow $ DC1 vs DC2, stress1 vs stress2)
Series2DiffCond	Extracts all ‘DCs’ from a ‘series’
Collector	Combines several tokens of the same type to a list
Distributor	Extract the individual tokens from a list

**Figure 2:**
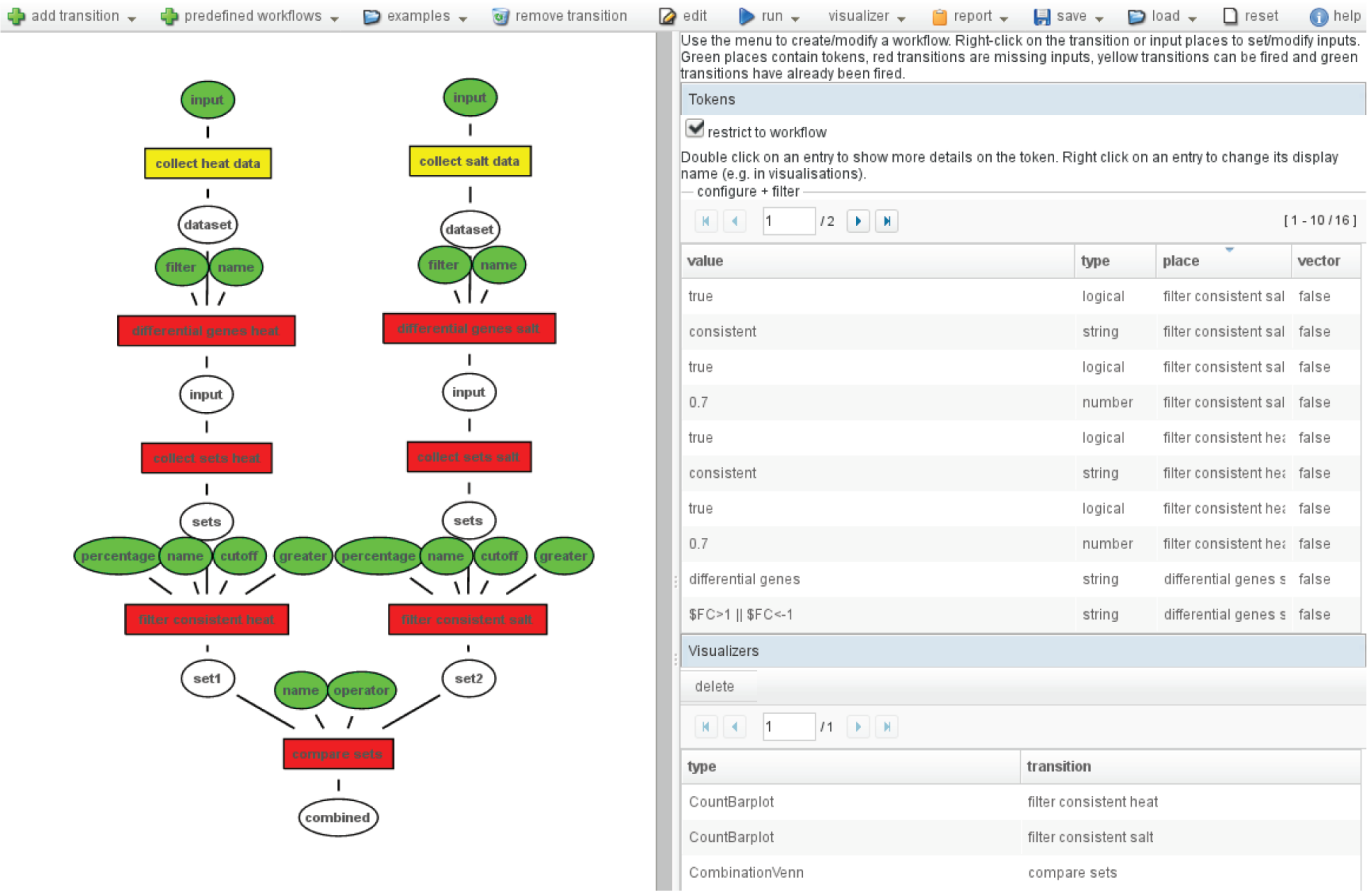
Example workflow: On the left, the Petri net workflow comparing the consistent genes between heat and salt stress is shown. On the right, an overview of all tokens used in the workflow is shown. The color of the inputs (ellipses) indicates whether it contains a token (green), and the color of the transitions (boxes) shows whether it has been fired (green), can be fired (yellow) or cannot be fired because input tokens are missing (red).

These transitions can be connected to elaborated workflows. Figure 2 shows an example workflow. It consists of multiple transitions that can also depend on each other, i.e. the output of one transition is used as input for another transition. These workflows can be executed automatically, or single transitions are selected for execution. Executing single transitions allows one to interactively evaluate the results of the transition and modify the inputs if necessary before executing the subsequent steps from the workflow.

The tokens in the workflow system can have several types: ‘DC’, ‘series’, ‘set’, ‘DAG’ and ‘network’ as well as the simple types ‘string’, ‘boolean’ and ‘number’. To allow for transitions that have a variable number of inputs or outputs of the same type (e.g. to calculate the intersection of the differential genes from several ‘DCs’), we introduce the notion of token lists, which are simply lists of tokens of the same type. There are helper transitions to combine several tokens to a list token or to isolate the individual tokens from a list token.

The initial tokens can be extracted from the ‘DC’ and (time/concentration) ‘series’ contained in the database. Additionally, the ‘DAGs’ and the corresponding ‘sets’ of the gene ontology (9) and different kinds of ‘networks’ for yeast, such as Yeastract (20), BioGRID (4), post-translational modification networks (6, 7, 19) and manually curated stress networks (13) are available.

### Interactive report

**Figure 3:**
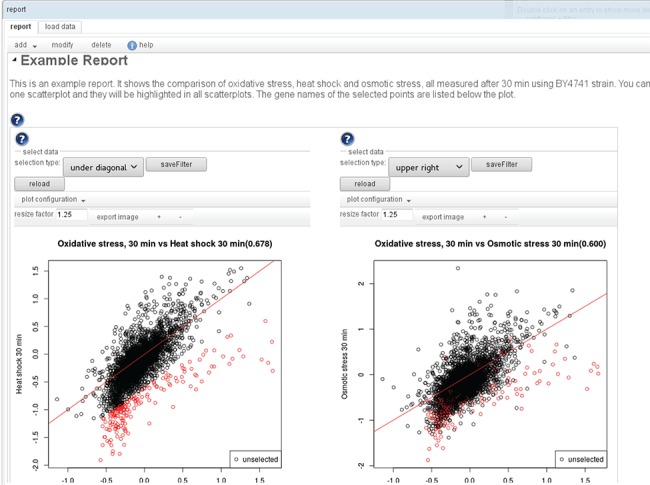
An example report: A report consists of sections, with text and interactive visualizations. An editor allows one to modify/add/delete the individual elements and to change their order. Many of the available visualizations are also interactive. E.g. in the scatterplot shown here, sets of genes can be selected to highlight them (also in other subplots of the visualization) and to display the labels of the selected points in a list below the plot.

The result of a workflow is not only the final output, but intermediate results can be just as interesting. To provide a convenient way to get an overview of all the results, the user can add to each transition one or more visualizers. When the workflow is executed, the visualizers generate plots, tables or network views that are all added to one report (see Figure 3). For most transitions, there are standard visualizers, but additionally, the user can also define custom visualizations to be included in the report, by defining the plot type and inputs. Most visualizations are interactive, so that the associated data of points in a plot or rows in a table can be retrieved. In the example in Figure 3, the genes selected in the left scatterplot comparing oxidative stress and heat shock are not only listed below the plot but also highlighted in the right scatterplot comparing oxidative and osmotic stress.

The resulting report can be edited, by adding and removing sections, visualizations and descriptive text or changing the order of the elements. This way, a report that summarizes the results of the workflow is created. It can then be saved as an XML file, which can be uploaded to our website to show the report. This allows one to share the results with collaborators or to save intermediate results for later refinement.

If the workflow is executed again with different inputs, another report with the same visualizations using the new data is created. If the two runs of the workflow should be compared, a joined report that contains the results for both runs next to each other is produced. This allows the user to easily compare different parameterizations of the same workflow, e.g. to compare the effects of different cutoffs for the definition of differential genes or analyzing another type of stress.

## Results

Here we present an example analysis that compares the genes involved in two types of stress: heat shock and osmotic stress. Already in 2000, Gasch and Werner-Washburne (8) showed that yeast responds similarly to a wide range of different types of stress including heat shock and osmotic stress. They observed up- and downregulation of two clusters of genes, which they termed ‘environmental stress response’. Moreover, the survival of one type of stress can ‘cross-protect’ yeast cells from a different type of stress, as e.g. heat shock increases tolerance for osmotic stress (18). To analyze which genes are unique to the two types of stress and which are shared, we first identify the genes that are consistently changed for each type of stress, and then these two sets are compared to each other.

YESdb already contains a predefined workflow to define the consistently changed genes from a list of data sets, which can be added to an analysis. This predefined workflow selects for each of the data sets the changed genes and uses the ‘Count Filter’ transition to identify the genes that are changed in a given fraction of all data sets. The resulting set contains only those genes that are consistently changed and not those that are changed in only a few of the data sets, e.g. due to technical bias or strain-specific responses to the stress that do not capture the ‘core’ stress response. In the predefined workflows contained in YESdb, most inputs are already set to default values, and in this example only the data sets that should be analyzed have to be selected.

Using our selection interface, we can select all heat shock data sets measured at 37°C, exclude all data sets using knockout strains and restrict the selection to those data sets measured after 15 min, resulting in 19 data sets. Similarly, we can select those 10 osmotic stress data sets measured 30 min after 0.4M NaCl was added, which did not use knockout strains. Overall, 5627 and 3488 genes are changing (|fold change| >1) in at least one of the selected heat shock and osmotic stress data sets, respectively, of which 796 and 1770 are consistently changing in at least half of the selected data sets.

To compare the sets of consistently changed genes that are the result of the two copies of the predefined workflow, we add a ‘binary set combination’ transition. This transition applies a set operation (intersect, difference or union) to two given sets. Using this transition, we can define the set of genes that is unique for heat shock or osmotic stress, or the set of genes that are shared between the two types of stress. There are 725 shared genes and 71 and 1045 genes unique to heat shock and osmotic stress, respectively. The resulting workflow is shown in Figure 2.

**Figure 4:**
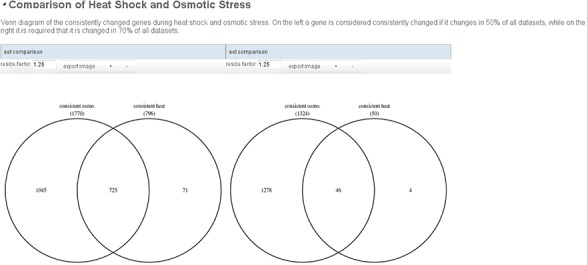
Example report comparing two runs of the example analysis. On the left, a strict cutoff for the definition of consistently changed genes is used (only genes that are changed in at least 70% of all data sets) while on the right genes that are changed in at least half of the data sets are considered. The resulting Venn diagrams are shown side by side so that the user can easily assess the different results of the analysis.

The inputs of this workflow can be varied to analyze the robustness of the results. We could e.g. change the cutoff above which percentage of data sets a gene has to change in to be considered consistent from 50% to 70%. This changes the number of consistently changing genes to 59 and 1324 in heat shock and osmotic stress, respectively. This shows that the selected heat shock data sets are less consistent than the osmotic stress data sets, maybe because the heat shock at 37°C is a very mild stress to which the different wild-type strains that were analyzed in the data sets do not react similarly. To visualize the results of such an comparative analysis, a report comparing multiple runs of the same workflow can be created. Figure 4 shows such a report. Similarly to the normal report it contains headers and descriptive text and visualizations. Visualizations that are automatically created from visualizers added to transitions are shown for all selected runs of the workflow, side by side. This way the different results can easily be analyzed.

The example presented here is only one of many possible analyses. It can easily be extended to e.g. characterize the resulting gene set further by gene set enrichment. Similar analyses can be used to tackle different questions like how different strains react to stress, how stress strength influences stress response or whether there is a platform bias.

## Discussion

Public databases like GEO, ArrayExpress or SRA contain thousands of data sets that often measure similar experimental conditions. Combining these data sets provides more robust results because technical biases and noise can be removed. If different biological entities like proteins and gene expression are measured, the integration provides a more complete picture of the changes in the cell. Moreover, different experimental conditions can be compared to identify shared mechanisms.

The stress response system in the model organism *S. cerevisiae* is a well-studied system that is nevertheless not completely understood. There are measurements for different kinds of stress, different strengths, different time frames and on different experimental platforms. The integration of these data sets can help to understand the exact changes in response to a single stress and shared and divergent mechanisms between different kinds of stress.

YESdb is a database that contains over 3000 DCs of yeast stress measurements using microarray, next-generation sequencing and proteomics platforms. It combines the yeast stress-related data sets of GEO, ArrayExpress, SRA and PRIDE and provides access to already preprocessed data on the level of DCs. The data sets are annotated to different kinds of stress, publication years, platforms and strains. An easy-to-use interface is used to select the relevant data sets for further analysis.

A Petri net-based workflow system allows one to combine a given set of transitions to elaborated analyses that identify and combine interesting sets of genes and characterize them. Even though these transitions correspond to quite simple operations, the possibility to combine them in any way allows not only to perform standard analyses but also specialized analyses for a given research question.

The results of such an analysis can be visualized in an interactive report. For most transitions, visualizers can be added to the workflow that will automatically add a visualization of the result of the transition to the report. This can be especially useful to compare different runs of the same workflow that differ in some parameter. The resulting report contains the visualizations side by side so that the effect of the changed parameter on the results of the various steps in the workflow can be easily analyzed. Additionally, the user can create own visualizations by selecting plot type and inputs from all available inputs and (intermediate) results. Many of the visualizations are interactive, e.g. tables are sortable or information about individual points in a plot can be shown. To explain the results and structure the report, text and subsections can be added to the report, so that a human-readable report of the analysis can be created. The report can be saved as an XML file and uploaded to our website to show the report, so that reports can be shared e.g. between collaboration partners.

The annotation contained in YESdb provides a valuable resource for systematic analyses. It can be used to systematically analyze differences between platforms, strains, types of stress or the strengths of the applied stress. Furthermore, it contains data sets for 203 knockout strains that can be used to compare the effects of the knockout in different types of stress or to understand the regulatory mechanisms in general.

While this system is now only available for stress response in yeast, we think that also other research topics can benefit from this system. To use the interactive workflows and reports for another biological system, the corresponding data sets have to be identified, processed and annotated to create the underlying database. Additionally, the set of available transitions can be extended to include also more complex operations.

## Notes

**Database URL:** https://services.bio.ifi.lmu.de/YESdb
